# The prevalence and burden of recurrent headache in Australian adolescents: findings from the longitudinal study of Australian children

**DOI:** 10.1186/s10194-021-01262-2

**Published:** 2021-06-01

**Authors:** Margot J. Wilkes, M. Dilani Mendis, Leanne Bisset, Felix T. Leung, Christopher T. Sexton, Julie A. Hides

**Affiliations:** 1 School of Health Sciences and Social Work, Griffith University, Nathan, Brisbane 4111 Australia; 2 Physiotherapy Department, Mater Health Services, South Brisbane, 4101 Australia; 3 The Hopkins Centre, Menzies Health Institute Queensland, Griffith University, Gold Coast, 4222 Australia; 4 The University of Queensland, School of Dentistry, Herston, Brisbane 4006 Australia

**Keywords:** Prevalence, Recurrent headache, Adolescents, Health-related quality of life, Longitudinal study of Australian children

## Abstract

**Background:**

Headache disorders are highly prevalent worldwide, but not well investigated in adolescents. Few studies have included representative nationwide samples. This study aimed to present the prevalence and burden of recurrent headache in Australian adolescents.

**Methods:**

The prevalence of recurrent headache, headache characteristics (severity and frequency) and burden on health-related quality of life in Australian children aged 10–17 years were presented, using nationally representative data from the Longitudinal Study of Australian children (LSAC). The LSAC, commencing in 2004, collects data every 2 years from a sample of Australian children of two different age cohorts: B ‘baby’ cohort, aged 0–1 years and K ‘kindergarten’ cohort, aged 4–5 years at the commencement of the study. Face-to-face interviews and self-complete questionnaires have been conducted with the study child and parents of the study child (carer-reported data) at each data collection wave, with seven waves of data available at the time of the current study. Wave 7 of the LSAC was conducted in 2016, with B cohort children aged 12–13 years and K cohort children aged 16–17 years. For the current study, data were accessed for four out of seven waves of available data (Wave 4–7) and presented cross-sectionally for the two cohorts of Australian children, for the included age groups (10–11 years, 12–13 years, 14–15 years and 16–17 years). All available carer-reported questionnaire data pertaining to headache prevalence, severity and frequency, general health and health-related quality of life, for the two cohorts, were included in the study, and presented for male and female adolescents. Carer-reported general health status of the study child and health-related quality of life scores, using the parent proxy-report of the Paediatric Quality of Life Inventory™ 4.0, were compared for male and female adolescents with recurrent headache and compared with a healthy group. Finally, health-related quality of life scores were compared based on headache frequency and severity.

**Results:**

The LSAC study initially recruited 10,090 Australian children (B cohort *n* = 5107, K cohort *n* = 4983), and 64.1% of the initial sample responded at wave 7. Attrition rates across the included waves ranged from 26.3% to 33.8% (wave 6 and 7) for the B cohort, and 16.3% to 38.0% (wave 4–7) for the K cohort. Recurrent headache was more common in females, increasing from 6.6% in 10–11 years old females to 13.2% in 16–17 years old females. The prevalence of headache in males ranged from 4.3% to 6.4% across the age groups. Health-related quality of life scores were lower for all functional domains in adolescents with recurrent headache, for both sexes. Headache frequency, but not severity, was significantly associated with lower health-related quality of life scores, in both males and females.

**Conclusions:**

Recurrent headache was common among Australian adolescents and increased in prevalence for females, across the age groups. Frequent recurrent headache is burdensome for both male and female adolescents. This study provides information regarding the prevalence and burden of recurrent headache in the adolescent population based on findings from the Longitudinal Study of Australian Children.

## Background

Headache is one of the most common disorders of the nervous system and can cause substantial levels of disability [1]. The epidemiology of headache disorders was collated in 2007, with a global prevalence of 46% reported for adults with general headache [1]. The longitudinal Global Burden of Disease Survey confirmed that headache disorders are among the top 10 causes of disability worldwide [2] and that migraine is the second leading cause of years lived with disability in adults [3]. Headache disorders are a major public health problem worldwide, can affect quality of life and are associated with impairments in daily functioning, lost productivity, economic burden and reduced social interaction in adults [4–6]. While it is known that the long-term prognosis of headaches is often poor [7, 8] and that headache in earlier years of life is associated with headache in adulthood [9], knowledge regarding the prevalence and burden of headache prior to adulthood, such as during the adolescent years, remains limited.

Adolescence is the phase of development between childhood and adulthood, from ages 10 to 19 years [10]. It is a crucial period of time in the natural history of some primary headache disorders, however, there are few population-based studies on headache prevalence during this period of development [5]. Previous surveys conducted in different countries and cultures have reported considerable variation in the prevalence of headache disorders in adolescents, with values ranging from 30% up to 88% in school-based studies [11–13]. In this age group, headache disorders can cause school absenteeism, participation restrictions and reduced social interaction, consequently delaying educational progress and imposing a burden likely to be expressed throughout many domains of life [11, 13–16]. The prevalence of recurrent headache in the Australian adolescent population is currently not known, nor has the impact of recurrent headache on health-related quality of life (HRQoL) been investigated in this population.

While there are some data regarding the prevalence of headache in adults, these data cannot necessarily be applied to adolescents. In order to further inform health care professionals about factors contributing to the development, persistence, and health-related disability of headache over time, information regarding the prevalence and burden of headache during the adolescent years is required. Furthermore, it has been recognised that aggregation of data into wide age bands in epidemiological studies of adolescent headache may not adequately capture variation across the adolescent years, and the burden across narrow age bands is currently unknown. Therefore, using data from Growing Up in Australia: The Longitudinal Study of Australian Children (LSAC), our study aimed to document the prevalence and characteristics of recurrent headache in two nationally representative cohorts of Australian adolescents, using narrow age bands. Secondly, our study aimed to compare HRQoL between adolescents with and without recurrent headache, using a carer-reported general health scale and the Paediatric Quality of Life Inventory (PedsQL™4.0) parent proxy-report scale. Finally, our study aimed to investigate the impact of headache frequency and severity on HRQoL in Australian adolescents with recurrent headache.

## Methods

Data were obtained from the LSAC, a nationally representative study of Australian children, conducted conjointly between the Australian Department of Social Services, the Australian Institute of Family Studies and the Australian Bureau of Statistics. For the current study, data were presented cross-sectionally for four out of seven waves of available data (Wave 4–7), from two different aged cohorts of Australian children.

### LSAC study design, participants and procedure

The LSAC, commencing in 2004, has biennially followed two representative samples of Australian children. Detailed information on the LSAC study design, participant inclusion criteria, questionnaire development and field methods have been reported previously in the literature [17, 18]. Briefly, the LSAC used a dual-cohort clustered sampling design, with Medicare Australia enrolment as the sampling frame and Australian postcodes as primary sampling units. Children aged 0 to 1 years (born between March 2003 and February 2004; B cohort) and children aged 4 to 5 years (born between March 1999 and February 2000; K cohort) were randomly selected using the database of Medicare, Australia’s national health care system in which 98% of Australian children are enrolled by 12 months [19]. Postcodes were stratified by state then randomly selected to represent urban/rural distributions, to ensure proportional numbers of children (according to population statistics) were randomly selected within each postcode. The sample in the first year was intended to be representative of Australian children in each of the two selected age cohorts, and included Australian children deemed as citizens, permanent residents and applicants for permanent residency. Response rates to the initial mailed out invitation in 2004 (18,814 families initially contacted) were 57.2% for the B cohort (5107 infants) and 50.4% for the K cohort (4983 children) [17]. Succeeding data waves have been collected every 2 years from this sample, subject to attrition from non-response or non-contact, including loss of contact, participant opting out (refusal and where the study child is deceased), or the child moving beyond the scope of collection (moving overseas). Some children are brought back into the sample if contact can be re-established [17]. Lower response rates were observed for Aboriginal or Torres Strait Islander children, and for children from non-English-speaking backgrounds [20].

At each wave, face-to-face interviews and self-complete questionnaires have been conducted with the study child and their parent/s [18, 21]. Further information on development of the questionnaires can be found online, in the LSAC Data User Guide [21]. The LSAC was approved by the Australian Institute of Family Studies review board, and written informed consent was provided by a caregiver of each child [18].

### Current study design, participants and procedure

For the current study, a cross-sectional design was used. At the time of this study, seven waves of data were accessible from the LSAC. Data were drawn from Wave 6 and 7 for the B cohort (to include male and female children aged 10–13 years), and from Wave 4, 5, 6 and 7 for the K cohort (to include male and female children aged 10–17 years), so as to represent the Australian adolescent population. Only carer-reported data were included in this study. For the purpose of this study, Parent 1 data was used and defined as ‘carer-reported’ data. Parent 1 is deemed by the LSAC to be the parent that knows the most about the study child, and is most often the child’s biological mother, but may also be the child’s father or guardian [18, 21]. All available Parent 1 carer-reported data for male and female children in the included age groups, and for the chosen study measures were included in the current study [18, 21].

### Measures

Carer-report measures for headache across the chosen waves included recurrent headache as an ongoing condition, headache frequency and headache severity. In the LSAC administered questionnaire, the term ‘frequent’ headache was used for the B cohort, while ‘recurrent’ headache was used for the K cohort. Regardless of cohort, recurrent/frequent headache was defined in the questionnaire as ‘being one that exists for some period of time (weeks, months, years) or reoccurs regularly’. The term ‘recurrent’ headache will be used for the remainder of this paper, for both cohorts. Headache prevalence was established by carer-reported response to ‘Does child have any of these ongoing conditions: recurrent headaches (yes/no)?’ Headache frequency and severity were established by carer-reported response to the questions: ‘How often do recurrent headaches occur [for the study child] (daily; most days, being 4-6 times a week; some days, being 1-3 times a week; a few times a month; or rarely)?’ and ‘Would you describe the child’s recurrent headaches as mild, moderate or severe?’

Carer-reported HRQoL was assessed using a five-point general health scale and the PedsQL™ 4.0 parent proxy-report. The general health scale required the carer to indicate ‘in general, how would you say the child’s current health is?’ on a five-point Likert scale (excellent, very good, good, fair, poor). The PedsQL™ 4.0 is a commonly used measure of paediatric HRQoL and has been evaluated in several paediatric chronic illness samples [22]. The PedsQL™ 4.0 has demonstrated good internal consistency (α child = 0.88, α parent = 0.90) in healthy children and children with acute and chronic illness, including headache [22–24]. Parent proxy-reports are available for children aged 8–18 years and assess parents’ perceptions of their child’s HRQoL. Child self-report PedsQL™ 4.0 scales are not included in the LSAC, for the included waves.

The PedsQL™ 4.0 parent proxy-report yields a total HRQoL score and two summary scores, calculated from four Generic Core Scales: physical functioning (eight items), emotional functioning (five items), social functioning (five items), and school functioning (five items). To obtain the Physical Health Summary Score, the physical functioning Generic Core Scale is used. To calculate the Psychosocial Health Summary Score, items in the emotional, social and school functioning scales are used and a mean score is computed. A 5-point response Likert scale (0 = never a problem, 1 = almost never a problem; 2 = sometimes a problem; 3 = often a problem; 4 = almost always a problem) is utilised, in response to how much of a problem each item has been for the study child during the past 1 month, within each functioning Generic Core Scale. Items are reverse scored and linearly transformed to a 0–100 scale (0 = 100, 1 = 75, 2 = 50, 3 = 25, 4 = 0), so that higher scores indicate better HRQoL. Scale scores are computed as the sum of the items divided by the number of items answered, to account for missing data [22, 25].

### Statistical analysis

The confidentialised Growing Up in Australia: LSAC Release 7.2, April 16, 2020 (Waves 1–7) was accessed under the Murdoch Children’s Research Institute license from Dataverse (https://dataverse.ada.edu.au/dataverse/lsac). Child sample weights, specific to each cohort and wave, were applied to compensate for differences between the sample and the target national population arising from non-response and sample attrition; those who were more likely to respond, had decreased weighting [20]. Analysis was undertaken using Stata (version 14.2 StataCorp, Texas, USA, 2016) and survey commands were used due to the stratification sampling by state regions, clustering by postcode and to apply the sample weights.

Demographic specific information was collected for each age group and cohort. Study child characteristics for each age group are presented as percentages with 95% confidence intervals (CI) or mean (CI). Family demographics including country of birth, Indigenous status, spoken language at home if other than English, if the family resides in a greater capital city, and the Index of Relative Socio-economic Advantage (SEIFA) data were collected from the included waves and cohorts were combined. The study child’s sex, body mass index, and stages of puberty are presented for each age group and cohort. Carer-reported general health data were collected from the included waves, and cohorts combined.

In addressing our first aim, the estimated prevalence of recurrent headache was calculated at the included waves, for the two cohorts separately, and presented for males and females, for the included age groups. The headache frequency and severity analysis was based on the subset of participants who reported recurrent headache, with sample weights applied, and presented as percentage and 95% CIs. Headache frequency was grouped into: ‘rarely/few times per year’, ‘few times each month/some days of the week’, or ‘most days of the week/daily’. Headache severity was classified as mild, moderate or severe. For both headache frequency and severity, data were collapsed across the two cohorts as estimates were similar for the two cohorts. A Pearson chi-square test, adjusted for the survey design, was used to assess the independence of the proportions of frequency or severity of headache across the age groups.

For our second aim, the proportion of male and female adolescents within each general health category (excellent, very good, good, fair or poor) were presented for those that had a carer-reported response of ‘yes’ to recurrent headache, compared to adolescents with a carer-reported response of ‘no’ to recurrent headache. Data were collapsed across cohorts.

PedsQL™ 4.0 parent proxy-report Generic Core Scale scores (physical, emotional and social functioning), the Psychosocial Health summary score and a total HRQoL score were compared between those with recurrent headache (‘recurrent headache and associated conditions’ group) and a ‘healthy’ group. The ‘recurrent headache and associated conditions’ group allowed for the inclusion of those adolescents that had a carer-reported response of ‘yes’ to recurrent headache, and a carer-reported response of ‘yes’ to ‘Does the child have any of these ongoing conditions? Hayfever, recurrent abdominal pain, recurrent pain in other parts of the body, bone/joint/muscle problem, Attention Deficit (Hyperactivity) Disorder, anxiety disorder, depression, chronic fatigue, recurrent chest pain, recurrent back pain?’. An ongoing condition was defined as one that exists for some period of time (weeks, months or years) or re-occurs regularly, and the study child did not have to be diagnosed by a doctor. Adolescents with the following ongoing conditions were excluded from the ‘recurrent headache and associated conditions’ group: eczema, asthma, vision problems, ear infections, hearing problems, tonsillitis, diarrhoea/colitis, constipation, soiling, wetting self during the day, autism, Asperger’s or other autism spectrum, diabetes, epilepsy or seizure disorder, other infections, other illnesses. The ‘healthy’ group included adolescents that had a carer-reported response of ‘no’ to recurrent headache and ‘no’ to all listed ongoing conditions. Survey adjusted linear regression modelling and post-estimation marginal contrasts (F-test) were used to estimate and assess the domains of HRQoL between the recurrent headache group and healthy group, while controlling for age. Finally, HRQoL Generic Core Scale scores (physical, social and emotional functioning) the Psychosocial Health summary score and a total HRQoL score were compared by headache frequency and severity groups, for the subset of adolescents that had a carer-reported response of ‘yes’ to recurrent headache, using a survey adjusted Wald F-test of coefficient equality. Significance was set at *p* < 0.05.

## Results

### Demographics and characteristics of the study population

The number of children who responded to the LSAC at Wave 1, attrition rate, and sample size for the included waves, and cohorts in the current study, are presented in Table 1. Demographic information including puberty status and general health ratings are presented in Table 2, for the B and K cohort and for each age group. The proportion of males and females was similar across the two cohorts. The majority of the children were born in Australia, did not identify as being Aboriginal or Torres Strait Islander, English was the primary language spoken at home and the respondents lived in urban locations.

**Table 1:** Cross-sectional response and attrition rate for the included waves, age groups and cohorts

	Wave 1 (2004)	Wave 4 (2010)	Wave 5 (2012)	Wave 6 (2014)	Wave 7 (2016)
**B cohort**					
Age group (years)	0–1	–	–	10–11	12–13
^a^Attrition rate (%)	–	–	–	26.3	33.8
Study child, male (n)	–	–	–	1929	1734
Total (n)	5107	–	–	3764	3381
**K cohort**					
Age group (years)	4–5	10–11	12–13	14–15	16–17
^a^Attrition rate (%)	–	16.3	20.6	29.0	38.0
Study child, male (n)	–	2132	2020	1798	1576
Total (n)	4983	4169	3956	3537	3089

^a^Cross-sectional attrition rate, those not responding to that particular wave, as a percentage of the Wave 1 cross-sectional response with data taken from LSAC Technical Paper No. 20 [20]. This table refers to the number of children who responded at each included wave

**Table 2:** The baseline characteristics (percentage and 95% CI) of study children by age group

Sample characteristic of the study child	Age group			
	10 to 11	12 to 13	14 to 15	16 to 17
**Male**				
*B cohort*	51.3 (49.6, 53.0)	51.0 (49.1, 52.9)		
*K cohort*	51.2 (49.6, 52.8)	51.8 (50.0, 53.5)	51.4 (49.7, 53.2)	51.4 (49.3, 53.5)
Born in Australia*	97.9 (97.5, 98.3)	98.0 (97.6, 98.4)	96.2 (95.4, 96.9)	96.1 (95.1, 96.8)
ATSI*	3.8 (3.2, 4.5)	3.2 (2.6, 3.9)	2.6 (2.1, 3.4)	2.8 (2.1, 3.8)
^**a**^Language*	10.5 (8.9, 12.4)	9.9 (8.2, 11.8)	10.2 (8.5, 12.2)	9.5 (7.8, 11.4)
Greater capital city*	64.0 (62.3, 65.6)	63.6 (61.9, 65.2)	62.9 (60.8, 64.9)	62.4 (60.1, 64.7)
^**b**^SEIFA*	1009.4 (1003.4, 1015.4)	1005.0 (998.8, 1011.3)	1007.0 (1000.7, 1013.3)	1003.7 (996.9, 1010.5)
**Body mass index**				
*B cohort*				
Underweight	6.8 (5.9, 7.8)	6.5 (5.5, 7.6)		
Normal weight	67.2 (65.3, 69.1)	66.7 (64.7, 68.6)		
Overweight or obese	26.0 (24.4, 27.7)	26.9 (25.0, 28.8)		
*K cohort*				
Underweight	5.9 (5.1, 6.8)	6.5 (5.7, 7.4)	6.3 (5.5, 7.3)	6.5 (5.5, 7.6)
Normal weight	65.5 (63.6, 67.4)	65.7 (63.8, 67.5)	65.1 (63.2, 66.9)	62.3 (60.1, 64.4)
Overweight or Obese	28.6 (26.8, 30.4)	27.8 (26.1, 29.6)	28.6 (26.9, 30.4)	31.3 (29.2, 33.4)
**Puberty, female study child**				
Menstruation commenced				
*B cohort*	7.7 (6.3, 9.4)	51.7 (48.6, 54.7)		
*K cohort*	5.7 (4.5, 7.2)	57.0 (54.7, 59.3)	94.6 (93.3, 95.7)	98.8 (97.7, 99.3)
Breast growth				
*B cohort*	42.5 (39.7, 45.3)	80.7 (78.3, 82.9)		
*K cohort*	43.3 (40.9, 45.9)	81.1 (79.1, 83.0)	87.0 (85.2, 88.7)	93.6 (91.9, 94.9)
**Puberty, male study child**				
Voice deepened				
*B cohort*	2.3 (1.6, 3.3)	28.6 (26.0, 31.3)		
*K cohort*	1.5 (0.9, 2.4)	24.5 (22.6, 26.5)	75.4 (73.1, 77.5)	90.0 (88.1, 91.6)
Facial hair				
*B cohort*	1.5 (0.9, 2.3)	15.0 (13.0, 17.1)		
*K cohort*	0.9 (0.6, 1.5)	12.4 (10.8, 14.2)	49.4 (47.0, 51.8)	76.4 (73.9, 78.7)
General health*				
Excellent	43.3 (42.0, 44.7)	44.2 (42.6, 45.7)	44.5 (42.6, 46.3)	37.5 (35.4, 39.5)
Very Good	40.2 (39.0, 41.3)	39.8 (38.5, 41.1)	37.5 (35.8, 39.2)	41.1 (39.2, 43.1)
Good	13.8 (12.8, 14.5)	13.7 (12.8, 14.8)	13.9 (12.5, 15.4)	16.9 (15.4, 18.6)
Fair	2.4 (2.0, 2.9)	2.1 (1.7, 2.5)	3.3 (2.6, 4.1)	3.8 (3.1, 4.8)
Poor	0.3 (0.2, 0.5)	0.2 (0.1, 0.4)	0.9 (0.6, 1.3)	0.7 (0.4, 1.0)

Values represent % yes (CI)

*ATSI* Aboriginal or Torres Strait Islander, *SEIFA* Index of Socio-economic Advantage

*B and K cohort combined

^a^language other than English

^b^mean (CI)

### Carer-reported prevalence of recurrent headache

Table 3 shows the prevalence of recurrent headache for males and female for each cohort and age group. The prevalence of recurrent headache was similar between males and females in the 10–11 years age group but was consistently higher in females from 12 years of age onwards, for both cohorts. For the K cohort specifically, the prevalence of recurrent headache for females showed an increase with age from 6.6% to 13.2%, whereas for males in the K cohort, headache prevalence ranged from 4.3% to 6.4%.

**Table 3:** Carer-reported prevalence (percentage and 95% CI) of headache for males and females, by cohort and age group

Age (years)	Wave 4	Wave 5	Wave 6	Wave 7
**Male**				
10–11	6.4 (5.4, 7.6)		***4.6 (3.7, 5.8)***	
12–13		6.2 (5.2, 7.4)		***5.2 (4.0, 6.6)***
14–15			4.3 (3.4, 5.6)	
16–17				6.1 (5.0, 7.5)
**Female**				
10–11	6.6 (5.5, 7.9)		***5.4 (4.2, 6.8)***	
12–13		7.4 (6.2, 8.8)		***7.3 (5.8, 9.0)***
14–15			8.7 (7.3, 10.3)	
16–17				13.2 (11.5, 15.0)

Bold italics = B cohort

### Recurrent headache frequency and severity

Headache frequency for both males and females was dependent on age group (*p* = 0.013 and *p* = < 0.001 respectively). For both sexes, the majority of adolescents with recurrent headache reported experiencing headache a few times each month to some days of the week. The proportion of adolescents with recurrent headache of this frequency type, decreased across the age groups for both males and females. With increasing age, the proportion of adolescents reporting headache rarely/a few times per year increased (Table 4).

**Table 4:** Carer-reported headache frequency (percentage and 95% CI) for adolescents with recurrent headache, collapsed across cohorts

	Headache frequency			
	Rarely/ A few times per year	Few times each month/ Some days of the week	Most days of the week / Daily	
**Age (years)**				***p***
**Male**				
10–11 (*n* = 207)	9.9 (6.3, 15.3)	78.6 (71.7, 84.1)	11.6 (7.5, 17.4)	0.013
12–13 (*n* = 205)	16.3 (11.2, 23.0)	77.7 (70.3, 83.7)	6.1 (3.0, 11.8)	
14–15 (*n* = 71)	21.6 (13.0, 33.8)	68.4 (55.6, 78.9)	10.1 (4.9, 19.5)	
16–17 (*n* = 94)	27.3 (18.5, 38.1)	62.2 (50.9, 72.3)	10.5 (5.3, 19.7)	
**Female**				
10–11 (*n* = 218)	13.6 (9.2, 19.7)	81.4 (74.7, 86.7)	5.0 (2.7, 9.2)	< 0.001
12–13 (*n* = 246)	22.7 (16.9, 28.5)	71.8 (65.1, 77.7)	6.2 (3.4, 10.8)	
14–15 (*n* = 141)	17.5 (11.4, 25.9)	72.4 (63.6, 79.8)	10.1 (6.0, 16.5)	
16–17 (*n* = 191)	36.9 (29.7, 44.7)	54.5 (46.8, 62.1)	8.6 (4.7, 15.3)	

Headache of mild and moderate severity were more commonly reported than severe headache, across both sexes. A greater proportion of males and females reported moderate headache, in the 14–15 years age group (Table 5). The distribution of headache severity for females was dependent on age group (*p* = < 0.001). For males, there was not enough evidence that headache severity was dependent on age group (*p* = 0.077).

**Table 5:** Carer-reported severity of headache (percentage and 95% CI) for adolescents with recurrent headache, collapsed across cohorts

	Headache severity			
	Mild	Moderate	Severe	
**Age (years)**				***p***
**Male**				
10–11 (*n* = 207)	39.4 (31.9, 47.4)	37.1 (30.2, 44.6)	23.5 (18.2, 29.9)	0.077
12–13 (*n* = 205)	44.4 (37.4, 51.7)	38.8 (31.9, 46.2)	16.8 (11.8, 23.3)	
14–15 (*n* = 71)	25.8 (15.4, 39.8)	47.4 (34.6, 60.5)	26.9 (17.8, 38.3)	
16–17 (*n* = 94)	51.0 (38.7, 63.1)	30.2 (20.7, 41.8)	18.8 (10.4, 31.6)	
**Female**				
10–11 (*n* = 218)	51.9 (44.4, 59.4)	36.4 (29.5, 43.9)	11.7 (7.9, 17.0)	< 0.001
12–13 (*n* = 246)	54.1 (46.5, 61.5)	34.6 (28.4, 41.4)	11.3 (7.6, 16.5)	
14–15 (*n* = 141)	25.9 (18.6, 34.9)	51.2 (42.0, 60.3)	22.9 (16.1, 31.5)	
16–17 (*n* = 191)	40.5 (33.0, 48.5)	41.7 (33.9, 49.9)	17.8 (12.1, 25.3)	

### Carer-reported general health

**Fig. 1:**
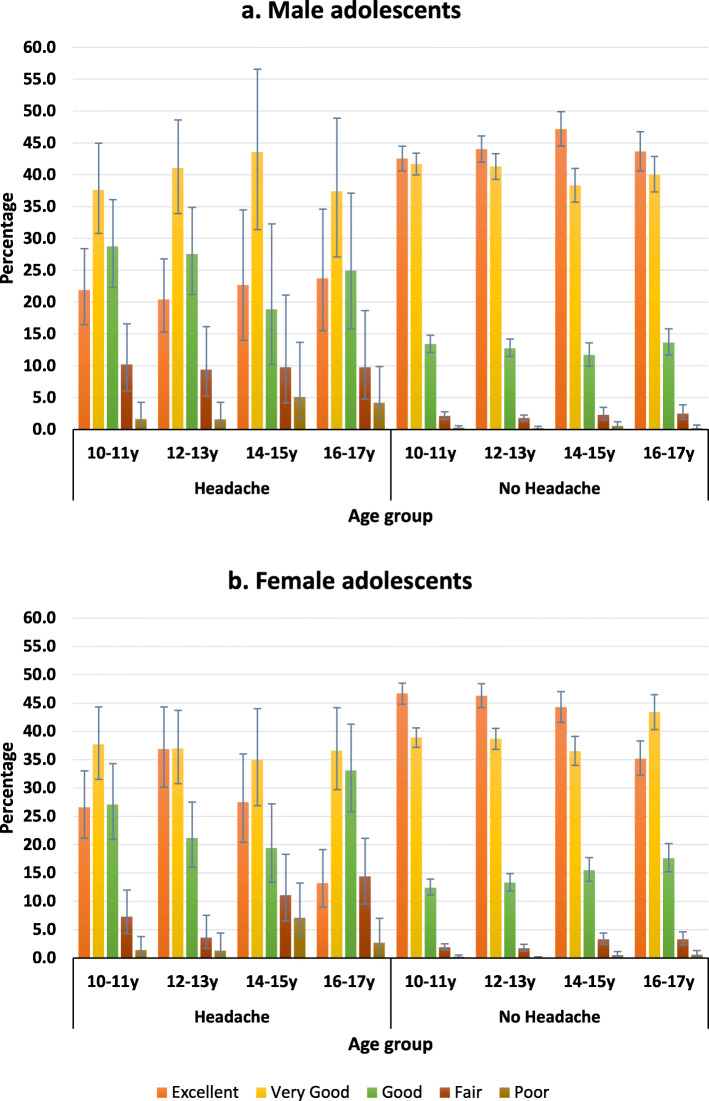
Carer-reported general health for males **a** and females **b** with and without recurrent headache. The carer-reported general health status of adolescents, collapsed across cohort, for those with a carer-reported response of ‘yes’ to [study child has] recurrent headache as an ongoing condition (male *n* = 577, female *n* = 795) and for those with a carer-reported response of ‘no’ to [study child has] recurrent headache as an ongoing condition (male *n* = 10,439, female *n* = 9737). The general health scale asked the carer to indicate on a five-point Likert scale, ‘in general, how would you say your child’s current health is (excellent, very good, good, fair, poor)?’. A greater proportion of adolescents with recurrent headache were classified as having fair to poor general health by their carer, compared to those without recurrent headache

Carer-reported general health was generally ‘poorer’ for both male and female adolescents with recurrent headache, compared to those without headache. A greater proportion of adolescents with recurrent headache were classified as having fair to poor general health by their carer, compared to those without recurrent headache. In males aged 16–17 years, 9.8% of adolescents with recurrent headache were classified as having ‘fair’ general health compared to 2.5% of male adolescents without recurrent headache (Fig. 1A). In females within this same age group, 14.4% of adolescents with recurrent headache were classified as having ‘fair’ general health, compared to 3.3% of female adolescents without recurrent headache (Fig. 1B).

### Carer-reported health related quality of life

Carer-reported HRQoL scores were significantly lower for all domains of the PedsQL™4.0, in males and females with recurrent headache and associated conditions, when compared with a healthy group. The only domain not to show a significant difference in HRQoL was social functioning in females aged 12–13 years (*p* = 0.10). However, a lower HRQoL score was still evident in this age group in female adolescents with recurrent headache and associated conditions (Table 6).

**Table 6:** HRQoL scores (mean and 95% CI) for males^a^ and females^b^ with recurrent headache and associated conditions, compared to a healthy group, collapsed across cohorts

PedQL domain	Age	Male			Female		
		Healthy group (no conditions)	Headache and associated conditions group	***p***	Healthy group (no conditions)	Headache and associated conditions group	***p***
Physical functioning	10–11	82.5 (81.5, 83.5)	74.0 (69.4, 78.5)	< 0.001	81.5 (80.4, 82.5)	76.1 (70.8, 81.5)	0.06
	12–13	85.2 (84.3, 86.1)	77.1 (73.2, 80.9)	< 0.001	85.1 (84.2, 86.0)	76.7 (73.2, 80.1)	< 0.001
	14–15	83.9 (82.2, 85.6)	67.9 (58.5, 77.3)	0.001	82.3 (80.7, 83.8)	71.9 (66.0, 77.8)	< 0.001
	16–17	85.1 (83.2, 87.0)	78.4 (73.6, 83.2)	0.013	82.9 (81.2, 84.6)	75.5 (71.3, 79.6)	0.002
Emotional functioning	10–11	77.0 (76.2, 77.8)	67.7 (63.5, 71.9)	< 0.001	76.5 (75.7, 77.3)	69.7 (65.9, 73.6)	< 0.001
	12–13	79.7 (78.9, 80.5)	69.4 (65.2, 73.5)	< 0.001	78.4 (77.5, 79.3)	67.4 (64.2, 70.7)	< 0.001
	14–15	81.5 (80.4, 82.6)	68.3 (62.5, 74.0)	< 0.001	78.0 (76.7, 79.2)	64.8 (59.4, 70.2)	< 0.001
	16–17	81.1 (79.7, 82.6)	60.0 (50.3, 69.7)	< 0.001	75.7 (74.2, 77.1)	63.5 (58.7, 68.3)	< 0.001
Social functioning	10–11	83.0 (82.0, 84.0)	77.43 (73.4, 81.3)	0.007	81.4 (80.4, 82.5)	77.2 (73.1, 81.2)	0.041
	12–13	85.1 (84.2, 86.0)	79.1 (74.4, 83.7)	0.013	84.3 (83.4, 85.2)	81.5 (78.2, 84.7)	0.10*
	14–15	84.3 (82.9, 85.7)	71.5 (63.0, 80.0)	0.003	81.3 (80.0, 82.6)	75.2 (69.7, 80.6)	0.036
	16–17	87.9 (86.2, 89.6)	80.7 (74.6, 86.9)	0.027	84.8 (83.2, 86.4)	81.8 (76.2, 87.5)	0.045
Psychosocial health summary score	10–11	78.4 (77.6, 79.1)	70.4 (66.9, 74.0)	< 0.001	79.2 (78.4, 79.9)	74.2 (70.8, 77.6)	< 0.004
	12–13	79.8 (79.1, 80.5)	71.0 (67.1, 74.8)	< 0.001	80.7 (79.9, 81.4)	74.5 (72.0, 77.2)	< 0.001
	14–15	79.4 (78.4, 80.4)	68.0 (62.3, 73.8)	< 0.001	78.7 (77.5, 79.8)	69.4 (64.8, 74.1)	< 0.001
	16–17	81.6 (80.2, 82.9)	67.2 (60.9, 73.6)	< 0.001	79.1 (77.8, 80.5)	72.0 (67.1, 76.8)	0.007
Total score	10–11	79.8 (79.0, 80.6)	71.7 (68.3, 75.0)	< 0.001	80.0 (79.2, 80.8)	74.9 (71.2, 78.6)	0.008
	12–13	81.7 (81.0, 82.4)	73.1 (69.6, 76.6)	< 0.001	82.2 (81.5, 83.0)	75.3 (72.7, 77.9)	< 0.001
	14–15	81.0 (79.8, 82.1)	68.0 (61.4, 74.6)	< 0.001	79.9 (78.8, 81.1)	70.3 (65.5, 75.0)	< 0.001
	16–17	82.8 (81.4, 84.2)	71.1 (65.9, 76.3)	< 0.001	80.4 (79.1, 81.8)	73.2 (69.1, 77.3)	0.001

*Non-significant

^a^Males (n): 10–11 years: Healthy group = 1967, Headache group = 98

12–13 years: Healthy group = 1935, Headache group = 97

14–15 years: Healthy group = 796, Headache group = 29

16–17 years: Healthy group = 602, Headache group = 35

^b^Females (n): 10–11 years: Healthy group = 1900, Headache group = 96

12–13 years: Healthy group = 1819, Headache group = 144

14–15 years: Healthy group = 702, Headache group = 67

16–17 years: Healthy group = 519, Headache group = 74

A significant difference in HRQoL scores for all domains was observed in both males and females with recurrent headache, based on headache frequency (Table 7). Lower HRQoL scores were reported by those with headache that occurred ‘few times month/some days of the week’, and lower again for those with headache that occurred ‘most days/daily’. However, no significant difference in HRQoL scores were seen in males and females based on headache severity group (*p* > 0.05).

**Table 7:** HRQoL scores (mean and 95% CI) for males and females with recurrent headache, by headache frequency and severity

	Headache frequency				Headache severity			
PedQL domain	Rarely	Few times month/some days	Most days/daily	***p***	Mild	Moderate	Severe	***p***
**Male (*n*** **= 568)**								
Physical functioning	77.0 (72.1, 82.0)	71.5 (69.1, 74.0)	64.3 (58.5, 70.0)	0.005	71.7 (68.9, 74.6)	71.9 (68.6, 75.3)	71.5 (67.2, 75.7)	0.99
Emotional functioning	66.9 (61.9, 72.0)	62.1 (59.5, 64.7)	50.5 (41.9, 59.2)	0.008	61.9 (58.8, 65.1)	61.7 (58.2, 65.2)	61.7 (56.6, 66.8)	0.99
Social functioning	76.2 (71.6, 80.8)	72.3 (69.5, 75.1)	59.0 (50.7, 67.3)	0.002	72.7 (69.2, 76.2)	70.7 (66.5, 74.9)	71.4 (66.7, 76.0)	0.76
Psychosocial health summary	69.2 (64.5, 74.0)	64.7 (62.2, 67.2)	54.2 (47.5, 60.9)	0.002	64.5 (61.4, 67.5)	64.0 (60.6, 67.4)	65.2 (61.0, 69.4)	0.87
Total	72.0 (67.5, 76.5)	67.1 (64.8, 69.4)	57.7 (51.6, 63.8)	0.001	67.0 (64.3, 69.7)	66.8 (63.7, 69.9)	67.4 (63.5, 71.4)	0.96
**Female (*n*** **= 782)**								
Physical functioning	77.8 (75.0, 80.6)	72.4 (70.4, 74.4)	69.6 (63.0, 76.2)	0.005	73.3 (71.1, 75.5)	73.2 (70.5, 75.8)	74.5 (70.3, 78.7)	0.85
Emotional functioning	67.9 (64.7, 71.0)	61.0 (58.8, 63.2)	55.2 (49.8, 60.7)	< 0.001	63.1 (60.5, 65.7)	60.0 (57.4, 62.6)	64.9 (60.9, 69.0)	0.06
Social functioning	80.3 (76.7, 83.9)	73.8 (71.6, 76.0)	70.7 (64.6, 76.8)	0.005	75.1 (72.5, 77.7)	73.8 (71.3, 76.3)	78.0 (73.9,82.0)	0.16
Psychosocial health summary	73.7 (70.7, 76.7)	67.4 (65.5, 69.4)	61.1 (56.7, 65.5)	< 0.001	68.8 (66.6, 71.1)	66.9 (64.7, 69.1)	71.1 (67.6, 74.5)	0.09
Total	75.1 (72.4, 77.9)	69.2 (67.4, 71.0)	64.0 (59.7, 68.4)	< 0.001	70.4 (68.4, 72.4)	69.1 (67.0, 71.2)	72.3 (69.0, 75.4)	0.23

## Discussion

This study presented data from a large nationally derived sample of Australian adolescents, aged 10 through to 17 years of age. The results of the current investigation showed that the prevalence of recurrent headache varied both by sex and age group. Recurrent headache prevalence was similar for both males and females in the 10–11 years age group. After this, female adolescents showed a progressive increase in recurrent headache prevalence across the age groups, up to 13.2% (CI 11.5, 15.0) in 16–17 years old females. For adolescent males, recurrent headache prevalence was seen to be more consistent over the age groups and ranged from 4.3% to 6.4%.

While our data are not specific to migraine, two systematic reviews of migraine have shown that in early adolescence, males and females are equally likely to be affected by migraine, but by late adolescence the prevalence of migraine is higher in females, with a ratio similar to that seen in adults [26, 27]. Female reproductive hormones are known to be associated with increased risk of migraine, and the prevalence of migraine has been shown to be greater in females after the age of menarche [28, 29]. In our study, the prevalence of recurrent headache increased in females over the age groups, in line with an increased percentage of females in each age group reporting that menstruation had commenced. Previous studies of headache prevalence in males have shown that the prevalence of headache is relatively stable throughout puberty [28]. For migraine, males tend to be diagnosed with migraine at a younger age, with peak age onset between 10 and 14 years. Thereafter, the male dominance of migraine gives way to the female predominance seen in adults [30], with a similar pattern demonstrated in our study results. Remission of migraine has been shown to occur in 18–34% of adolescents, with evidence that early aggressive interventions may result in disease modification [30]. Of note in our study, a drop in the prevalence of recurrent headache was demonstrated in 14–15 years old males (K cohort). While it is not known what causative factors may have contributed to this lower prevalence of headache in this age group, it has been suggested that testosterone may play a role in migraine remission [31]. Therefore, further understanding of recurrent headache prevalence, and possible associations with age, stage of puberty and fluctuations in sex hormones, is an important area for further research, and may improve our understanding of disease modification and headache management across the adolescent years.

Our study further investigated the frequency and severity of recurrent headache in the Australian adolescent population. Our results showed a significant association between headache frequency and age group, for both males and females with recurrent headache. In females, headache frequency changed from being predominately a ‘few times each month/some days of the week’ in those aged 10–15 years, to ‘rarely/a few times per year’ in 16–17 years old females. This change in headache frequency in this age group could possibly be due to pubertal stage. Previous population-based studies have shown that menarche increases the risk of headache, however longitudinal analyses did not reveal an increase in headache frequency after menarche [32, 33]. Males showed a similar change in headache frequency by age group, but the change was not as evident as that seen in females. For headache severity, an increase in the percentage of females with moderate or severe recurrent headache was seen in the 14–15 years age group, with a reduced percentage reporting mild headache. No significant difference in headache severity across age groups was reported in males. The shift to more severe headaches at 14–15 years of age in females may again be due to pubertal stage and commencement of menarche. Our findings showed that commencement of menarche was documented in 95% of 14–15 years old females in the K cohort, an increase from 57% in the previous age group. Our findings provide a unique insight into the changing characteristics of recurrent headache across the adolescent years.

While several studies have examined the impact of headache on the quality of life of adults [34–36], this is only an emerging area of research in adolescents [23, 24]. Firstly, our study showed lower HRQoL scores across all domains of the PedsQL™4.0 using parent proxy-report, for those with recurrent headache and associated conditions, compared to a healthy group. Similar findings have been presented in children with migraine headache, using the child and parent-proxy reports of the PedsQL™4.0 [24]. Powers, Patton [24], in their clinic-based study, found that children with migraine headache reported lower HRQoL scores than children in a healthy comparison sample. Parent report of HRQoL for children in the migraine headache group was also significantly lower than parent report in the healthy sample. Additionally, correlations between child and parent proxy-report for the summary and total scores of the PedsQL™4.0 were found to be statistically significant, and correlations were in the medium to large effect size range [24]. The findings of our study, using parent proxy-report, is therefore likely to reflect the impact of recurrent headache on the functional and emotional wellbeing of adolescents, within the Australian population. HRQoL is an important construct to assess, especially as it relates to treatment effectiveness and patient satisfaction [37, 38]. Furthermore, while self-report is considered the standard for measuring perceived HRQoL, it is often the parents’ perceptions of their children’s HRQoL that influences healthcare utilization [25]. Finally, our study showed that the frequency of headache, but not severity, was associated with lower HRQoL scores across all domains, in both males and females. This novel finding directs the need for further research into the impact that headache frequency and severity has on HRQoL and may aid the clinician in identifying those individuals with recurrent headache that may be at risk of experiencing a greater disease burden and hence further direct healthcare utilization.

The current investigation presented headache prevalence in two nationally representative cohorts of Australian adolescents, from the LSAC. The LSAC has many strengths including its complex sample design, sample size, representative cohort and strong retention of participants allowing for population inferences to be made [20]. Furthermore, the inclusion of survey commands (stratification sampling by state regions, clustering by postcode) and sample weights applied to the current study, allows for more accurate inferences to be made from the sample frequencies to the population [20], therefore the estimates in the current study are likely to be reliable beyond this sample and apply to the population from which the sample came. The relationship between recurrent headache, and the headache characteristics of frequency and severity, and impact on HRQoL, within a strong representative sample of Australian adolescents, has not previously been explored. The current investigation provides robust evidence of both the extent of recurrent headache experienced in Australian adolescents at different ages across the adolescent years, and the burden of recurrent headache on HRQoL. There are however some limitations to our study. Firstly, all outcomes in our study were based on carer-reported data. Carer-reported data have the potential for underreporting of headache prevalence, severity and frequency. Carer-reported data were used in our study to ensure consistency in outcome measures across the included waves. Carer-reported data remain an important perspective of a child’s health and may assist in directing health care focus over the adolescent years. Secondly, trends in the prevalence of recurrent headache for the included age groups in our study indicated an association with the stage of puberty and onset of menarche. However, only limited data were presented on pubertal stage for the age groups in this study. Research on the influence of puberty and sex hormone fluctuations on recurrent headache in Australian adolescents is an area of important future investigation, however, was beyond the scope of this study. Finally, HRQoL scores were taken from the parent proxy-report from the PedsQL™ 4.0, as child self-reports were not available for the included waves. While the parent proxy-report scales represent only the parents’ perception of the child’s HRQoL, the scales were constructed to directly parallel the child self-report items [25]. Furthermore, a moderate degree of concordance has been demonstrated previously between child and parent proxy-report scores of children/adolescents with migraine headache using the PedsQL™ 4.0 [23, 24], and with trends shown towards higher intercorrelations with increasing age in the pediatric HRQoL literature [39]. Thus, parent proxy-report remains a useful indication of the burden of recurrent headache in the Australian adolescent population.

## Conclusions

Australian adolescents aged 10–17 years showed trends in recurrent headache prevalence based on age and sex, similar to trends previously observed in studies of migraine sufferers. An increasing proportion of female adolescents reported recurrent headache over the included age groups, from age 10 through to 17. This increase is in line with previous findings of migraine prevalence and stage of puberty in females. The prevalence of recurrent headache in males was more consistent over the included age groups. Headache frequency and severity were also influenced by age group and sex. HRQoL scores were lower for all domains of the PedsQL™ 4.0 in adolescents with recurrent headache and associated conditions, compared with those without any reported medical conditions. Finally, headache frequency, but not headache severity, was significantly associated with lower HRQoL scores for all domains in both males and females. Findings from this population-based prevalence study may direct healthcare utilization and treatment prioritisation for male and female adolescents.

## Data Availability

The data that support the findings of this study are available from Growing up in Australia: The Longitudinal Study of Australian Children, at https://growingupinaustralia.gov.au/data-and-documentation. General release data can be access via application to National Centre for Longitudinal Data (NCLD) Dataverse at https://dataverse.ada.edu.au/dataverse/ncld.

## Abbreviations

LSAC: Longitudinal Study of Australian Children
B cohort: Baby cohort
K cohort: Kindergarten cohort
HRQoL: Health-related quality of life
PedsQL™4.0: Paediatric Quality of Life Inventory™ 4.0
ATSI: Aboriginal or Torres Strait Islander
SEIFA: Index of Socio-economic Advantage
CI: Confidence interval
